# Examinations of effects of socio-demographic features and disease-related data of patients with hemodialysis on the quality of life

**DOI:** 10.1038/s41598-023-43473-4

**Published:** 2023-10-02

**Authors:** Elif Togay, Hatice Öntürk Akyüz

**Affiliations:** 1 Department of Dialysis, Bitlis Tatvan State Hospital, Bitlis, Turkey; 2 Nursing Department, Faculty of Health Sciences, Bitlis Eren University, Rahva Campus, Beş Minare District, Ahmet Eren Boulevard, 13100 Center/Bitlis, Turkey

**Keywords:** Medical research, Nephrology

## Abstract

This study aimed to detect the effects of demographic features and disease-related data of individuals with hemodialysis treatment on quality of life. The research universe consisted of 113 patients who received dialysis treatment in three state hospitals. Sampling was not used in this research with 97 volunteer patients who complied with the study criteria. The data collection process was performed face-to-face between 15 March and 15 April 2021. Data was collected using the Participant Information Form and the Quality of Life Scale (Short From-36). In our research, 50.5% of participants were male and 49.5% were female. The age of 61.9% of participants was ≥ 51 years and 78.4% were married. Most participants (67%) had ≥ 3 children, 35.1% received dialysis treatment for 3–10 years, 90.7% had dialysis three times a week, and 64.9% had an arterio-venous fistula vascular access line. In addition, 24% had another family member who received dialysis treatment, with hypertension as the etiology in 35.6% of chronic renal failure (CRF). The findings showed that 93.8% patients had no previous transplantation, and 62.9% considered future transplantation.Within the scope of this research, the findings suggest that the quality of life of dialysis patients was poor. Demographic features and disease data affected the quality of life at different levels.

## Introduction

CRF has increased in recent years worldwide as well as in our country, Turkiye. CRF has been a significant public health problem that causes morbidity and mortality. CRF is defined as a functional disorder of progressive type that develops due to nephron damage, fluid electrolyte imbalance, and regression in renal metabolic and hormonal functions. The global prevalence of CRF is thought to range from 9 to 12%^1–4^. CRF incidence increases with age and about 38% of the estimated CRF population is > 65 years of age^5^. CRF is defined as kidney damage or decreased and/or loss of renal function expressed as at least three (3) months of GFR (glomerular filtration rate) without considering any reason^6^. CRF is classified into five stages according to the severity of the disease and its symptoms^2,3^.

The health-related quality of life-HRQoL of patients with CRF is worse than that of the general population in both early and advanced stages of CRF^3,6–8^. Most HRQoL-related studies are of cross-sectional type. Most studies have focused on dialysis patients. However, the interpretation of any HRQoL report depends on the validity and reliability of the tools^9,10^.

The dialysis process is one of the CRF treatment methods. However, this treatment decreases the quality of life and increases the patient’s dependence. In recent years, there have been so many trials to provide and maintain the quality of life and daily comfort of patients on dialysis treatment. The quality of life is measured with different scales. The most frequently used the quality of life scales in the field of health care are The Kidney Disease The Quality of Life Short Form and Short Form Health Survey Questionnaire (SF-36)^11^.

When determining quality of life, the degree of an individual’s level of activity in physiological, psychological, and sociological fields and level of satisfaction with economic status should be considered. There are four facets of SF-36 quality of life. These are psychological/mental health, physical health, social and personal health, and financial and economic health. The existence of the chronic disease, being in grief, anger, helplessness, hopelessness, worry, being in a constant urge to cry, tides in family relations, loss of functional role activities in work life, loss of self-esteem, change of body image, depressive appearance, concern about dependence and fear of death are the other factors that decrease the quality of life^8,11,12^.

An obligatory invasive treatment 3–4 days a week for an average of four hours makes patients’ lives dependent on the dialysis machine, health team and family and affects every aspect of their lives. In various studies, it was proven that the quality of life of patients on dialysis treatment is poor. In addition, various factors are known to negatively affect the quality of life of individuals on treatment negatively^13,14^. Our study aimed to detect the effects of socio-demographic features and disease-related data of individuals with hemodialysis treatment on the quality of life.

## Methods

This research was conducted in descriptive type. The research universe included a total of 113 patients who attended dialysis treatment at Bitlis State Hospital, Güroymak State Hospital and Bitlis Tatvan State Hospital. In this research, it was aimed to reach all universes. Thus, it was conducted with 97 volunteer patients who complied with inclusion criteria (90% of the universe). The data were collected from the participants by face-to-face interview method after the necessary institutional permissions were obtained by the researcher.

### Inclusion criteria

Individuals who were on dialysis treatment for at least three months, age range between 18 and 65 years, without any known psychological disorders, willing to participate voluntarily, and without any communication problems.

### Exclusion criteria

Individuals with any known psychological disorders and not giving consent for this research.

### Data collecting tools

Research data were collected with the “Participant Information Form” and “Short Form 36 (SH-36) Scale”.

### Participant information form

The participant Information Form was created by researchers parallel to the literature^15,16^. It contained two parts with 15 questions. In the first part, there were six questions about socio-demographic features (age, gender, educational status, marital status, number of children and family type). In the second part, there were nine questions and data about dialysis treatment (when it is started, frequency of sessions, line of vascular access, dialysis history in family members) were collected.

### SH-36 scale

Short form quality of life scale is the best-known and most commonly used health-related general quality of life scale in health care research. SF-36 quality of life scale is formed by Ware et al. (1992) and is made available for research^11^. Turkish validity and reliability were performed by Koçyiğit et al. in our country^12^. SF-36 is one of the scales used to evaluate physical and mental health to measure the quality of life. SF-36 evaluates an individual’s quality of life in the concept of general health without focusing on any age, disease or treatment group^8,11^. SF-36 is different from other scales by being a self-assessment scale, easy to fill by the patient himself in a short time, by evaluating not only negative but also positive aspects of health and by detecting minor changes in disability^17^. It is stated that SF-36 in dialysis patients could be used for research purposes and individual follow-up and considers last four week evaluations^16^. The confidence interval for analysis was set as 95%, and *p* < 0.05 was noted as statistically significant.

### Ethical consideration

To conduct this research was approved from Bitlis Eren University Ethics Committee (No: E.6266, Date: 05 March 2021). This study was conducted in accordance with the Declaration of Helsinki. Written approval was obtained from the required institution and informed consent was obtained from each participant. Informed consent was obtained from illiterate participants by reading the informed consent form verbally by the researcher and a legal guardian.

### Statistical analysis

Data obtained from this study were analyzed with the SPSS (Statistical Program in Social Sciences 25) program. Mann Whitney U test was used to compare scores obtained by the SF-36 quality of life Scale according to two-category variables (e.g., gender). Kruskal Wallis H test was used to compare scale scores according to more than two-category variables (e.g., age groups). Dual comparisons were made to detect the origin of the difference and Bonferroni correction was performed. In the scope of this research, the Shapiro–Wilk normality test was used to examine the distribution of scores obtained with the SF-36 quality of life scale. The confidence interval for analysis was set as 95%, and *p* < 0.05 was noted as statistically significant.

## Results

As shown in Table 1, 49.5% and 50.5% of participants were female and male, respectively. Most participants (61.9%) were in the ≥ 51 years of age group and 78.4% of participants were married. Most participants (67%) had ≥ 3 children. Also, 47.4% of participants lived in nuclear and 52.6% were in large families; 34% of the participants were illiterate, 15.5% were literate, and 30.9%, 17.5% and 2.1% were in elementary schools, high school and bachelor’s degree graduate, respectively.

**Table 1:** Distribution of participants according to demographic features.

	f	%
Gender		
Female	48	49.5
Male	49	50.5
Age		
≤ 30 years	10	10.3
31–40 years	13	13.4
41–50 years	14	14.4
≥ 51 years	60	61.9
Marital status		
Married	76	78.4
Single	18	18.6
Other	3	3.1
No	22	22.7
Number of children		
1 child	2	2.1
2 children	8	8.2
≥ 3 children	65	67.0
Family type		
Nuclear family	46	47.4
Large family	51	52.6
Illiterate	33	34.0
Literate	15	15.5
Educational status		
Elementary school graduate	30	30.9
High school graduate	17	17.5
Bachelor’s degree graduate	2	2.1

As shown in Table 2, 16.5% of participants received dialysis treatment for 0–1 year, 27.8% for 1–3 years, 35.1% for 3–10 years and 20.6% > 10 years. Most participants (90.7%) received dialysis three times a week. Thirty-two percent had a permanent tunneled catheter, 3.1% had a temporary tunneless catheter and 64.9% had arterio-venous fistula vascular access lines. In addition, 24% of participants stated that there was another family member who received dialysis treatment. Those who received treatment as a family member are more likely the first (46.4%) and second-degree (39.3%) relatives relatives. About eighty-five percent of participants claimed they know about CRF. In this study, according to participants, the etiology of CRF were hypertension (HT) (35.6%), others (31.1%) and Diabetes Mellitus (DM) (25.6%) and 93.8% stated that they were not previous transplant recipients. Also, 62.9% considered a future transplantation (Table 2).

**Table 2:** Distribution of participants according to information about dialysis treatment.

	f	%
Duration of dialysis treatment		
0–1 year	16	16.5
1–3 years	27	27.8
3–10 years	34	35.1
10+ years	20	20.6
Frequency of weekly dialysis		
1 time a week	1	1.0
2 times a week	1	1.0
3 times a week	88	90.7
> 3 times a week	7	7.2
Vascular access line		
Permanent tunneled catheter	31	32.0
Temporary tunneless catheter	3	3.1
Arterio-venous fistula	63	64.9
Any other family member who received dialysis treatment?		
Yes	24	24.7
No	73	75.3
Individuals who received dialysis treatment in family *		
First degree relative	13	46.4
Second degree relative	11	39.3
Third degree relative	4	14.3
Do you know the etiology of CRF?		
Yes	83	85.6
No	14	14.4
The etiology of CRF*		
DM	23	25.6
HT	32	35.6
FMF	0	0.0
Inherited	7	7.8
Other	28	31.1
Previous transplantation?		
Yes	6	6.2
No	91	93.8
Have you considered transplantation?		
Yes	61	62.9
No	36	37.1

*CRF* chronic renal failure, *DM* diabetes mellitus, *HT* hypertension, *FMF* familial mediterranean fever.

*More than one option was marked for these questions.

Physical functioning, physical role function, emotional role function, and social functioning scores were not significantly different according to gender (*p* > 0.05). Nevertheless, energy/fatigue/vitality, mental health, pain and general health perception scores were significantly different according to gender (*p* < 0.05). Scores of energy/fatigue/vitality, mental health, pain and general health perception were significantly higher in males than females (Table 3).

**Table 3:** Comparison of SF-36 Quality of Life Scores according to Gender, Age, Marital Status, Number of Children, Family Type and Educational Status.

	Physical functioning	Physical role function	Emotional role function	Energy/ fatigue/ vitality	Mental health	Social functioning	Pain	General health perception
Gender								
Female	37.40 ± 22.1	14.58 ± 29.55	38.19 ± 21.73	31.67 ± 22.82	49.67 ± 22.94	47.66 ± 24.42	35.73 ± 34.61	25.52 ± 18.83
Male	44.8 ± 22.73	25.51 ± 36.62	34.69 ± 27.18	42.35 ± 22.57	62.04 ± 23.02	48.47 ± 27.79	52.76 ± 36.82	38.67 ± 21.69
	U = 943.5	U = −1.69	U = 1085	U = 839	U = 805.5	U = 1149.5	U = 844.5	U = 757
	*p* = 0.09	*p* = 0.09	*p* = 0.43	*p* = 0.01	*p* = 0.01	*p* = 0.85	*p* = 0.02	*p* = 0.00
Age*								
≤ 30 years	61.00 ± 19.83^a^	42.50 ± 39.18	52.50 ± 16.20	66.40 ± 22.96	66.40 ± 22.96	63.75 ± 28.53	73.00 ± 20.71	31.50 ± 15.10
31–40 years	56.54 ± 19.30^a^	23.08 ± 25.94	42.31 ± 25.46	60.92 ± 23.62	60.92 ± 23.62	50.00 ± 33.46	44.62 ± 38.62	39.62 ± 23.67
41–50 years	47.86 ± 19.29^a^	16.07 ± 28.77	33.93 ± 20.86	56.86 ± 23.44	56.86 ± 23.44	44.64 ± 23.88	47.50 ± 39.16	31.43 ± 24.37
≥ 51 years	32.92 ± 20.38^b^	16.67 ± 34.34	34.08 ± 23.46	52.87 ± 23.76	52.87 ± 23.76	45.83 ± 23.99	38.75 ± 36.00	30.83 ± 21.04
	H = 23.80	H = 7.04	H = 3.61	H = 7.45	H = 3.46	H = 3.88	H = 7.71	H = 1.61
	*p* = 0.00	*p* = 0.07	*p* = 0.31	*p* = 0.06	*p* = 0.33	*p* = 0.27	*p* = 0.05	*p* = 0.66
Marital status								
Married	38.42 ± 21.45	17.11 ± 32.71	35.09 ± 23.66	35.13 ± 22.45	55.05 ± 22.48	46.05 ± 24.60	41.09 ± 36.01	30.92 ± 21.00
Single	50.95 ± 24.48	30.95 ± 35.27	41.27 ± 27.7	44.05 ± 25.08	59.05 ± 28.03	55.36 ± 30.25	56.07 ± 37.04	36.67 ± 22.16
	U = 578.50	U = −1.72	U = 697.00	U = 616.50	U = 722.00	U = 614.00	U = 619.50	U = 671.00
	*p* = 0.05	*p* = 0.09	*p* = 0.29	*p* = 0.11	*p* = 0.50	*p* = 0.10	*p* = 0.12	*p* = 0.26
Number of children*								
No	56.14 ± 21.60^a^	36.36 ± 36.78^a^	39.39 ± 28.43	46.59 ± 23.77	62.00 ± 25.50	61.36 ± 29.86^a^	56.70 ± 34.84	38.41 ± 21.84
1–2 children	53.00 ± 23.94^a^	32.50 ± 47.21^a^	43.33 ± 31.62	37.00 ± 23.71	60.00 ± 19.96	45.00 ± 13.44^b^	51.75 ± 41.70	29.50 ± 20.34
≥ 3 children	34.23 ± 19.63^b^	12.69 ± 27.64^b^	34.36 ± 22.02	33.85 ± 22.39	53.23 ± 23.45	44.04 ± 24.91^b^	39.00 ± 35.68	30.46 ± 21.13
	U = 16.46	U = 8.62	U = 0.91	U = 5.33	U = 2.51	U = 6.82	U = 4.29	U = 2.74
	*p* = 0.00	*p* = 0.01	*p* = 0.63	*p* = 0.07	*p* = 0.29	*p* = 0.03	*p* = 0.12	*p* = 0.25
Family type								
Nuclear family	41.74 ± 19.16	25 ± 35.75	38.41 ± 27.19	38.91 ± 24.06	58.61 ± 23.49	48.1 ± 25.41	50.98 ± 37.87	31.74 ± 21.71
Large family	40.59 ± 25.51	15.69 ± 31.21	34.64 ± 22.07	35.39 ± 22.51	53.49 ± 23.84	48.04 ± 26.85	38.33 ± 34.64	32.55 ± 21.08
	U = 1064.00	U = 2316.00	U = 1092.50	U = 1072.00	U = 1035.50	U = 1145.00	U = 988.50	U = 1144.00
	*p* = 0.43	*p* = 0.11	*p* = 0.49	*p* = 0.46	*p* = 0.32	*p* = 0.84	*p* = 0.18	*p* = 0.83
Educational status*								
Illiterate	32.27 ± 18.07^a^	6.82 ± 21.90^a^	30.15 ± 22.83	49.58 ± 24.72^a^	49.58 ± 24.72^a^	45.08 ± 20.24	34.47 ± 35.39^a^	28.33 ± 20.45
Literate	37.33 ± 22.19^a^	5.00 ± 10.35^a^	28.67 ± 17.88	47.73 ± 20.03^a^	47.73 ± 20.03^a^	37.50 ± 25.00	28.33 ± 23.08^a^	26.33 ± 20.13
Elementary school graduate	41.33 ± 20.25^a^	33.33 ± 39.57^b^	43.17 ± 23.32	64.40 ± 22.85^b^	64.40 ± 22.85^b^	53.33 ± 26.25	47.25 ± 37.89^b^	39.00 ± 23.76
High school graduate & bachelor’s degree graduate	59.21 ± 24.51^b^	34.21 ± 39.27^b^	46.05 ± 22.95	60.00 ± 22.07^b^	60.00 ± 22.07^b^	53.29 ± 33.29	69.47 ± 33.27^b^	32.63 ± 17.67
	H = 16.79	H = 16.45	H = 0.07	H = 10.05	H = 8.72	H = 5.45	H = 14.37	H = 4.59
	*p* = 0.00	*p* = 0.00	*p* = 1.00	*p* = 0.02	*p* = 0.03	*p* = 0.14	*p* = 0.00	*p* = 0.20

*Groups with statistically significant differences between them were marked with upper symbol.

Physical role function, emotional role function, energy/fatigue/vitality, mental health, and social functioning pain and general health perception scores were not significantly different according to age groups (*p* > 0.05). However, physical function scores were significantly different according to age groups (*p* < 0.05). Physical function scores of participants in ≤ 30, 31–40 age and 41–50 age groups had significantly higher scores than ≥ 51 age group (Table 3).

Physical function, physical role function, emotional role function, energy/fatigue/vitality, mental health, and social functioning, pain and general health perception scores were not significantly different according to marital status ( *p* ≥ 0.05). Physical function, physical role function, emotional role function, energy/fatigue/vitality, mental health, social functioning, pain and general health perception were similar between married and single participants (Table 3).

Emotional role function, energy/fatigue/vitality, mental health, pain and general health perception scores were not significantly different according to the number of children (*p* > 0.05). Nonetheless, physical function, physical role function and social functioning scores were significantly different according to the number of children (*p* < 0.05). Physical function and physical role function scores of those without children and with 1–2 children were significantly higher than those with ≥ 3 children. Social functioning scores of those without children were significantly higher than those with 1–2, and ≥ 3 children (Table 3).

As shown in Table 3, physical function, physical role function, emotional role function, energy/fatigue/vitality, mental health, social functioning, pain and general health perception scores were not significantly different according to family type (*p* > 0.05). Participants of a nuclear and large family had a similar physical function, physical role function, emotional role function, energy/fatigue/vitality, mental health, social functioning, pain and general health perception.

It was understood that emotional role function, social functioning and general health perception scores were not significantly different according to educational status (*p* > 0.05). However, physical function, physical role function, energy/fatigue/vitality, mental health and pain scores were significantly different according to educational status (*p* < 0.05). The physical function score of high school & bachelor’s degree graduate participants was significantly higher than those of illiterate, literate and elementary school graduates. On the other hand, energy/fatigue/vitality, mental health and pain scores of high school & bachelor’s degree and elementary school graduates were significantly higher than those of illiterate and literate participants (Table 3).

When Table 4 is examined regarding duration of dialysis treatment, 16.5% of participants had 0–1 year, 27.8% had 1–3 years, 35.1% had 3–10 years and 20.6% had > 10 years. Most participants (90.7%) received dialysis three times a week. Thirty-two percent of participants had a permanent tunneled catheter, 3.1% had a temporary tunneless catheter and 64.9% had arterio-venous fistula vascular access lines.

**Table 4:** Comparison of SF-36 the quality of life scores according to participants’ descriptive features.

	Physical functioning	Physical role function	Emotional role function	Energy/fatigue/vitality	Mental health	Social functioning	Pain	General health perception
Duration of dialysis treatment								
0–1 year	42.19 ± 19.23	15.63 ± 31.46	43.75 ± 22.84	57.00 ± 28.38	57.00 ± 28.38	53.13 ± 27.20	47.19 ± 32.87	39.06 ± 22.38
1–3 years	41.11 ± 19.87	24.07 ± 38.90	39.26 ± 25.41	59.85 ± 24.27	59.85 ± 24.27	46.76 ± 27.65	56.67 ± 37.20	31.48 ± 19.89
3–10 years	42.65 ± 24.99	16.18 ± 28.12	34.12 ± 20.65	54.35 ± 21.69	54.35 ± 21.69	47.79 ± 24.90	32.65 ± 34.73	28.97 ± 20.63
10+ years	37.75 ± 25.47	25.00 ± 37.17	33.75 ± 24.76	52.40 ± 23.18	52.4 ± 23.18	46.25 ± 26.31	45.25 ± 38.05	33 ± 23.47
	H = 0.64	H = 1.03	H = 1.89	H = 2.94	H = 1.57	H = 0.67	H = 5.74	H = 2.61
	*p* = 0.89	*p* = 0.79	*p* = 0.59	*p* = 0.40	*p* = 0.67	*p* = 0.88	*p* = 0.12	*p* = 0.46
Frequency of weekly dialysis								
≤ 3 a week	41.28 ± 22.74	20 ± 33.56	37.41 ± 24.9	37.78 ± 22.98	56.67 ± 24.14	48.33 ± 25.71	43.81 ± 36.36	32.61 ± 21.22
≥ 3 a week	39.29 ± 22.44	21.43 ± 36.6	23.81 ± 16.27	27.86 ± 25.96	46.29 ± 14.76	44.64 ± 32.16	51.07 ± 41.6	26.43 ± 22.86
	U = 311.5	U = -0.38	U = 228	U = 224	U = 219	U = 314	U = 277.5	U = 251.5
	*p* = 0.96	*p* = 0.7	*p* = 0.15	*p* = 0.2	*p* = 0.18	*p* = 0.99	*p* = 0.6	*p* = 0.37
Vascular line*								
Permanent tunneled catheter	38.06 ± 21.24	15.32 ± 32.07	27.96 ± 17.42^a^	37.9 ± 26.13	57.29 ± 27.91	45.16 ± 30.56	44.84 ± 32.37	30.48 ± 20.79
Temporary tunneless catheter	43.33 ± 17.56	33.33 ± 57.74	66.67 ± 33.33^b^	36.67 ± 10.41	45.33 ± 6.11	70.83 ± 28.87	26.67 ± 26.73	35.00 ± 18.03
Arterio-venous fistula	42.54 ± 23.59	21.83 ± 33.45	39.15 ± 25.78^c^	36.67 ± 22.34	55.75 ± 22.01	48.41 ± 23.28	44.92 ± 39.06	32.86 ± 21.88
	H = 0.54	H = 1.79	H = 7.90	H = 0.07	H = 1.31	H = 1.86	H = 0.60	H = 0.31
	*p* = 0.77	*p* = 0.41	*p* = 0.02	*p* = 0.96	*p* = 0.52	*p* = 0.39	*p* = 0.74	*p* = 0.86
Dialysis treatment history in another family member?								
Yes	38.75 ± 22.9	20.83 ± 35.86	45.83 ± 23.70	32.29 ± 22.41	54.67 ± 27.17	42.71 ± 26.56	32.50 ± 35.02	25.00 ± 19.22
No	41.92 ± 22.62	19.86 ± 33.06	33.33 ± 24.22	38.63 ± 23.40	56.33 ± 22.63	49.83 ± 25.81	48.22 ± 36.46	34.52 ± 21.51
	U = 794.50	U = −0.07	U = 649.50	U = 731.00	U = 853.50	U = 764.00	U = 659.00	U = 639.50
	*p* = 0.49	*p* = 0.94	*p* = 0.02	*p* = 0.22	*p* = 0.85	*p* = 0.34	*p* = 0.07	*p* = 0.05
Do you know the etiology of CRF?								
Yes	42.17 ± 23.1	22.89 ± 35.4	36.95 ± 24.97	37.17 ± 22.21	56.87 ± 23.78	49.85 ± 25.04	45.84 ± 37.24	31.93 ± 20.97
No	35.00 ± 19.01	3.57 ± 9.08	33.33 ± 22.65	36.43 ± 29.38	50.29 ± 23.20	37.50 ± 30.22	35.36 ± 32.06	33.57 ± 23.81
	U = 486.00	U = −1.83	U = 533.00	U = 546.00	U = 484.00	U = 437.00	U = 486.00	U = 561.50
	*p* = 0.33	*p* = 0.07	*p* = 0.55	*p* = 0.72	*p* = 0.32	*p* = 0.13	*p* = 0.33	*p* = 0.84
Any previous transplantation?								
Yes	65.83 ± 25.77	37.50 ± 37.91	27.78 ± 25.09	45.83 ± 20.60	65.33 ± 18.01	66.67 ± 30.28	63.75 ± 33.72	29.17 ± 17.73
No	39.51 ± 21.57	18.96 ± 33.2	37.00 ± 24.57	36.48 ± 23.35	55.30 ± 23.97	46.84 ± 25.45	43.05 ± 36.56	32.36 ± 21.56
	U = 119.50	U = −1.68	U = 224.50	U = 202.00	U = 206.00	U = 173.50	U = 170.50	U = 252.50
	*p* = 0.02	*p* = 0.09	*p* = 0.38	*p* = 0.29	*p* = 0.31	*p* = 0.13	*p* = 0.12	*p* = 0.76
Do you consider future transplantation?								
Yes	47.62 ± 22.45	21.72 ± 34.3	33.88 ± 24.72	38.11 ± 20.19	57.51 ± 23.68	49.59 ± 25.82	49.26 ± 36.08	34.75 ± 20.20
No	30.14 ± 18.46	17.36 ± 32.63	40.74 ± 24.05	35.28 ± 27.80	53.22 ± 23.81	45.49 ± 26.58	35.97 ± 36.38	27.78 ± 22.6
	U = 590.00	U = −0.77	U = 928.50	U = 942.00	U = 947.00	U = 1004.50	U = 865.00	U = 863.00
	*p* = 0.00	*p* = 0.44	*p* = 0.13	*p* = 0.24	*p* = 0.26	*p* = 0.48	*p* = 0.08	*p* = 0.08

*CRF* chronic renal failure.

*Groups with statistically significant differences between them were marked with upper symbols.

According to Table 4, 24% of participants expressed that they had other family members receiving dialysis treatment. It was understood that other family members who received dialysis treatment were most likely first (46.4%) and second-degree (39.3%) relatives. The findings showed that 85.6% of participants expressed that they knew about CRF. Participants with CRF suggested that etiology was HT in 35.6%, other in 31.1% and DM in 25.6% of participants. About ninety-four percent had no previous transplantation history, and 62.9% considered future transplantation.

Physical function, physical role function, emotional role function, energy/fatigue/vitality, mental health, social functioning, pain and general health perception scores were not significantly different according to the duration of dialysis treatment (*p* > 0.05) as 0–1 year, 1–3 years, 3–10 years and 10+ years (Table 4).

Physical function, physical role function, emotional role function, energy/fatigue/ vitality, mental health, social functioning, pain and general health perception scores significantly different were not significantly different according to the frequency of weekly dialysis (*p* > 0.05). Participants who received dialysis in < 3 times a week and ≥ 3 times a week were similar regarding physical function, physical role function, emotional role function, energy/fatigue/vitality, mental health, social functioning, pain and general health perception (Table 4).

When Table 4 is examined, physical function, physical role function, energy/fatigue/vitality, mental health, social functioning, pain and general health perception scores were not significantly different according to the vascular line (*p *> 0.05). However, the emotional role function score was significantly different according to the vascular line (*p* < 0.05). The emotional role function score of participants with temporary tunneless catheter vascular lines was significantly higher than those with a permanent tunneled catheter vascular line.

Moreover, physical function, physical role function, energy/fatigue/vitality, mental health, social functioning, pain and general health perception scores were not significantly different according to the presence of a member of the family who received dialysis treatment (*p* ≥ 0.05). However, emotional role function scores were significantly different according to the presence of a member of the family who received dialysis treatment (*p* < 0.05). The emotional role function score of the participants who had other individuals who received dialysis treatment in their families was higher than those who did not have any individuals without treatment (Table 4).

As seen in Table 4, physical function, physical role function, emotional role function, energy/fatigue/vitality, mental health, social functioning, pain and general health perception scores were not significantly different according to the state of knowing the etiology of CRF (*p* > 0.05). Physical function, physical role function, emotional role function, energy/fatigue/vitality, mental health, social functioning, pain and general health perception were similar according to the state of knowing and not knowing the etiology of CRF.

As shown in Table 4, physical role function, emotional role function, energy/fatigue/vitality, mental health, social functioning, pain and general health perception scores were not significantly different according to the state of previous transplantation (*p* > 0.05). However, the physical function score was significantly higher according to the state of the previous transplantation ( *p* < 0.05).

Physical role function, emotional role function, energy/fatigue/vitality, mental health, social functioning, pain and general health perception scores were not significantly different according to the state of considering future transplantation (*p* > 0.05). However, the physical function score was significantly higher according to the state of considering future transplantation (*p* < 0.05).

## Discussion

CRF is a critical important life-threatening public health problem seen in almost every age group, that may cause loss of labor force and comorbidity, which leads to dependency on dialysis centers and health personnel, which affects the quality of life due to dialysis complications. Socio-demographic variables like gender, age, educational status and many children significantly affect the quality of life^18–22^.

In our research, 50.5% and 49.5% of participants were male and female, respectively. Gender is an important factor that affects the level of a patient’s quality of life^23^. The male gender had significantly higher energy/fatigue/vitality, mental health, pain and general health perception scores. In studies with findings consistent with our research, it was stated that male patients who received dialysis had higher quality of life^24^. However, there are several contradictory studies in the literature. In the research of Akyol Durmaz et al. (2016), it was reported that the level of quality of life was similar in male and female patients^14^.

Although several studies suggested a higher quality of life for female participants, to our knowledge, no study examined the reasons. Despite the lack of certain outcomes, the reasons for poor quality of life in women might be their intense role in domestic life and cultural and social structure. This might be associated with females’ poor options for nutrition and accommodation in Eastern societies, distress, anxiety and depression due to delivering many children. Women who have an intense role in domestic life should perform their duties whether they work or not. In addition, having a chronic disease makes life for women unbearable. Under all these circumstances, poor quality of life is inevitable^25^.

In our study, 61.9% of participants were ≥ 51 years of age. At the end of this research, it was seen that as age increased the average physical function score of the quality of life decreased. It was determined that the physical function scores of participants in the ≤ 30, 31–40 and 41–50 age range groups were higher than the ≥ 51 age group. In their study that examined the quality of life of patients with CRF(1), Atasoy et al.^1^ obtained similar findings. Kaner et al. examined the quality of life of hemodialysis patients and related factors, and they found that patients younger than 65 years had a lower quality of life^22^. There are many similar studies^26,27^.

Some studies have not revealed any relationship between age and quality of life. However, it has been suggested that age affects the quality of life^28^.

We shoul note that although age is an irreversible process, it is a state in which loss of physical function is at the forefront. Poor quality of life with increasing age is inevitable, keeping in mind the thought that as age advances various chronic diseases develop and physical restrictions accompany them^29^. We found consistent results in our research.

According to research results, 78.4% of participants were married. However, there was no significant relationship between the quality of life of being married and single. Similar and contradictory studies are present in the literature. In 2016, Durmaz Akyol^14^ stated that 72.4% of participants were married, and married participants had higher quality of life scores. However, Kring & Crane^30^ reported that there was no significant relationship between marital status and quality of life scores. This may be related to a woman’s place in society, cultural life and woman’s role.

The majority of participants (67%) stated that they had ≥ 3 children. Physical function and physical role function scores of those who did not have a child and had 1–2 children were significantly higher than those with ≥ 3 children. Social functioning scores of participants without children were significantly higher than those with 1–2, 3 and more children. In the literature, to our knowledge, there is no study about having children and the number of children. However, our study results indicated that the quality of life scores was higher in participants with children. This might be related to the thought that the presence of children provides a chance for care and support.

The findings showed that 34% of participants were illiterate. As an important factor that affects the quality of life, educational status has a direct impact on an individual’s self-development, having a job and the nature of the job. According to the research results, it was suggested that individuals with high educational status had higher income, more social opportunities and thus higher quality of life^29^. We had consistent results. Physical function scores of high school and bachelor’s degree graduate participants were significantly higher than those of illiterate, literate and elementary school graduates. Participants with high school, bachelor’s degrees and elementary school graduates had significantly higher energy/fatigue/vitality, mental health and pain scores than illiterate and literate participants.

According to research data of Bayın Donar, 37.8% of patients were high school and 35.4% were middle school graduates. 9.8% were illiterate^19^. In research, conducted in 2017 by the Department of Health Technology Evaluation, the findings showed that 52% of participants were middle school graduates and 23% were illiterate^31^. As a result of these studies, it was determined that patients’ educational status positively and significantly affected their adaptation to the disease course, their disease-related perceptions and levels of their quality of life^32^.

Data from our research indicates that there was no statistical relationship between the quality of life and the duration of dialysis. The findings showed that 35.1% of patients had been receiving dialysis treatments for 3–10 years. In Gökalp & Arpacı’s study with 324 patients, it was found that 50.6% of patients had been receiving dialysis treatment for six months to 1 year^32^. This findings suggests that the need for dialysis treatment is currently increasing. Together with an increasing number of patients in need of dialysis, a drop in the level of quality of life is inevitable. In our research, we found that 10 general scores of individuals with ≥ 10 years of dialysis treatments were low. There are similar and contradictory studies in the literature. In some studies, it has been suggested that there is no relationship between years of dialysis and quality of life scores^14^. However, in some other studies a relationship has been suggested^33^.

In our research, it was determined that 90.7% of participants had dialysis three times a week. Sub-scores of health status, the features related to hemodialysis, duration of hemodialysis treatments and frequency of weekly hemodialysis were similar. However, the hemodialysis access line affects the quality of life. It was found that 64.9% of participants had arterio-venous fistula vascular access lines. According to our research, emotional role function scores of participants with temporary tunneless catheter vascular lines were significantly higher than those with permanent tunneled catheter vascular line. There are a limited number of studies on this topic. Generally, in previous studies, it has been found that social function and general health perception sub-scores of those with arterio-venous fistula vascular access line are higher than those with a temporary or permanent catheter^19,33^.

In our research, 24% of participants had a family member who received dialysis treatment. The emotional role function score of participants who had another member in their families with dialysis treatment was significantly higher than those without any other family member who received treatment. In the literature, to our knowledge, there is no study indicating the quality of life of patients with a family member who received dialysis treatment. However, the result of our study could be interpreted as the presence of a family member who received dialysis treatment having some positive effects in terms of relieving feelings of loneliness, providing motivation, acting together, going to the dialysis center together and being better understood.

The findings showed that 93.8% of participants had no previous transplantation history and 62.9% considered future transplantation. Physical function scores of those with previous transplantation were significantly higher. Similarly, the physical function scores of those who considered transplantation were significantly higher. There are many studies in the literature that are consistent with our findings in the present study. In his study which problems suffered during the process of organ transplantation and the impact of these problems on the quality of life, Özşaker^34^ reported that the transplantation process is one of the important methods for renal failure and as a renal replacement treatment (RRT) transplantation increases the quality of life, and patients felt more confident and better^34^. In their study, Tung et al. reported that transplantation positively affects social and physical health and effective social support mechanisms positively affect the transplantation process^35^. Similarly, Kaya et al. stated that the majority of individuals start doing things they could not do before transplantation. Some could start a new job, and some young ones could return to school^36^.

Using research data, when the quality of life scores were calculated out of 100, the average score of the lowest perceived quality of life physical role function was 20.10 and the average general health perception score was 32.16. The average score for the highest mental health was 55.92 and the average score for social functioning was 48.07. When we considered all the research data, we found that the quality of life of participating dialysis patients was generally poor. However, it varied in different degrees according to various factors.

There are some limitations that should be considered in this study. Firstly, the data were collected from patients receiving hemodialysis treatment in a single rural province. The data are limited to the answers given by the patients who accepted to participate in the study to the questions in the demographic information form and the scale. Another limitation was that the religious and spiritual dimensions of life, which are known to have an intense impact on society and affect the quality of life, were not measured.

## Conclusions

CRF is a crucial life-threatening public health problem that is seen in almost every age group. It may cause loss of labor force and comorbidity, which leads to dependency on dialysis centers and health personnel. This study conducted in a province in the Eastern region of our country, Turkiye, indicated a poor quality of life. The main reasons are economic insufficiency, inadequate care and poverty. The purpose of dialysis treatment is not only to prolong life span but also to raise the level of quality of life. Adaptation to treatment course varies according to how the individual perceives the situation he/she is in, the communication and cooperation of team members (nurse, doctor, dietician, dialysis technician) with patients, and support from family and loved ones. When evaluating the quality of life, it is essential to bring the health status and variables about hemodialysis to the foreground, not ignore socio-demographic features like age, gender, family structure and educational status, and apply a holistic approach to raise the quality of life, use methods to raise the quality of life, evaluate the patient and disease-related factors in a holistic approach.

## Data Availability

The datasets generated or analyzed during this study are available from the corresponding author upon request.

## References

[1] Atasoy, İ, Çolak, H., Akdeniz, Y., Tanrısev, M. & Özyurt, B. Kronik böbrek yetmezliğinde yaşam kalitesi. *Tepecik Eğit. Hast. Derg.* **23**, 133–141 (2013).

[2] Gibertoni, D. *et al.* COVID-19 incidence and mortality in non-dialysis chronic kidney disease patients. *PLoS ONE* **16**, e0254525. 10.1371/journal.pone.0254525 (2021). 34242368 10.1371/journal.pone.0254525 PMC8270438

[3] Pagels, A. A., Soderkvist, B. K., Medin, C., Hylander, B. & Heiwe, S. Health-related quality of life in different stages of chronic kidney disease and at initiation of dialysis treatment. *Health Qual Life Outcomes* **10**, 3–11. 10.1186/1477-7525-10-71 (2012). 22710013 10.1186/1477-7525-10-71 PMC3511211

[4] Bikbov, B. *et al.* GBD Chronic Kidney Disease Collaboration. Global, regional, and national burden of chronic kidney disease, 1990–2017: A systematic analysis for the Global Burden of Disease Study 2017. *Lancet* **395**, 709–733 (2020). 10.1016/s0140-6736(20)30045-3. 10.1016/S0140-6736(20)30045-3 PMC7049905 32061315

[5] Varol, E. & Karaca Sivrikaya, S. Kronik böbrek yetmezliğinde yaşam kalitesi ve hemşirelik. *Düzce Üniv. Sağlık Bilimleri Enstitüsü Dergisi* **8**, 89–96 (2018).

[6] Scholl, L. F., Dickenmann, M. & Hirt-Minkowski, P. Outcome of dialysis patients aged seventy years or above: A retrospective analysis. *Swiss Med. Wkly.* **144**, w13920. 10.4414/smw.2014.13920 (2014). 24554289 10.4414/smw.2014.13920

[7] Seow, Y. Y., Cheung, Y. B., Qu, L. M. & Yee, A. C. Trajectory of quality of life for poor prognosis stage 5D chronic kidney disease with and without dialysis. *Am. J. Nephrol.* **37**, 231–238. 10.1159/000347220 (2013). 23467046 10.1159/000347220

[8] Selçuk, N. Y. Hemodiyaliz hastalarinda diyaliz yeterliliği ve yaşam kalitesi. *Turk. Klin. J. Nephrol-Spec. Top.* **8**, 6–11 (2015).

[9] Özdemir, Ü. & Taşcı, S. Kronik hastalıklarda psikososyal sorunlar ve bakım. *Erciyes Üniv. Sağlık Bilimleri Fakültesi Dergisi* **1**, 57–72 (2013).

[10] Kara, B. Ev hemodiyalizine yönelik inançlar ve deneyimler: Güncel kanitlarin gözden geçirilmesi. *Türk. Nefrol. Diyaliz Transplant. Dergisi* **25**, 17–23. 10.5262/tndt.03 (2016).

[11] Ware, J. E. Jr. & Sherbourne, C. D. The MOS 36-item Short-Form Health Survey (SF36). I. Conceptual framework and item selection. *Med. Care* **30**, 473–483 (1992). 1593914

[12] Koçyiğit, H., Aydemir, Ö., Ölmez, N. & Memiş, A. Kısa form-36 (KF36)’nın Türkçe versiyonunun güvenirliliği ve geçerliliği. *İlaç Tedavi Dergisi* **12**, 102–106 (1999).

[13] Pehlivan, F. *et al.* Kronik böbrek yetmezliği olan hastaların mizaç ve karakter özellikleri ve yaşam kalitesi. *Ordu Üniv. Tıp Dergisi* **3**, 13–16 (2016).

[14] Durmaz Akyol, A., Mertbilek, A., Kara, L. & Karadeniz, D. Arteriovenöz fistül kanülasyon işlemi sırasında kullanılan giriş tekniklerinin ağrı düzeyine olan etkisinin saptanması. *Nefrol. Hemşireliği Dergisi* **1**, 10–18 (2016).

[15] Keskinoğlu, P. & Çolakoğlu, S. Ölçeklerin farklı uygulama yöntemlerine göre tutarlılıklarının değerlendirilmesi. *Tepecik Eğit. Araşt. Hast. Dergisi* **26**, 58–62. 10.5222/terh.058 (2016).

[16] Erdoğdu, A., Sipahioğlu, N. T., Erginöz, E., Apaydın, B. & Sipahioğlu, F. Stapler hemoroidektominin yaşam kalitesine etkisinin SF-36 ölçeği ile değerlendirilmesi. *Ulusal Cer. Dergisi* **29**, 59–62. 10.5152/UCD.37 (2013). 10.5152/UCD.2013.37 PMC4379839 25931847

[17] Albayrak Okçin, F. & Usta Yeşilbalkan, Ö. Hemodiyaliz tedavisi alan kronik böbrek yetmezliği hastalarının yaşam deneyimlerinin incelenmesi. *Adıyaman Üniv. Sağlık Bilimleri Dergisi* **6**, 1–12. 10.30569/adiyamansaglik.608931 (2020).

[18] Hajian-Tilaki, K., Heidari, B. & Hajian-Tilaki, A. A comparison of health-related quality of life in patients with renal failure under hemodialysis and healthy participants. *Saudi J Kidney Dis. Transpl.* **28**, 133–140. 10.4103/1319-2442.198165 (2017). 28098114 10.4103/1319-2442.198165

[19] Bayin Donar, G. & Top, M. A conceptual framework of quality of life in chronic kidney disease in Turkey: A patient-focused approach. *Int. J. Health Plann. Mgmt.* **35**, 1335–1350. 10.1002/hpm.3038 (2020). 10.1002/hpm.3038 32744746

[20] Boz, E. & Topbaş, E. Ev hemodiyalizinde yaşanan uyku sorunları, yaşam kalitesi ve hemşirelik bakımı. *J. Nephrol Nurs.* **16**, 67–72. 10.47565/ndthdt.2021.34 (2021).

[21] Alhawatmeh, H., Alshammari, S. & Rababah, J. A. Effects of mindfulness meditation on trait mindfulness, perceived stress, emotion regulation, and quality of life in hemodialysis patients: A randomized controlled trial. *Int. J. Nurs. Sci.* **9**(2), 139–146 (2022). 35509694 10.1016/j.ijnss.2022.03.004 PMC9052255

[22] Kaner, G., Ayer, Ç. & Kaya, A. Ş. Determination of quality of life in patients on hemodialysis and evaluation of related factors. *Karya J. Health Sci.* **4**(1), 41–46. 10.52831/kjhs.1193747 (2023).

[23] Nguyen, N. T. Q. *et al.* Chronic kidney disease, health-related quality of life and their associated economic burden among a nationally representative sample of community dwelling adults in England. *PLoS One* **13**, e0207960. 10.1371/journal.pone.0207960 (2018). 30475893 10.1371/journal.pone.0207960 PMC6258125

[24] Mandoorah, Q. M., Shaheen, F., Mandoorah, S., Bawazir, S. & Alshohaib, S. Impact of demographic and comorbid conditions on qulity of life hemodialysis patients: A cross-sectional study. *Saudi J. Kidney Transpl.* **25**, 402–437. 10.4103/1319-2442.128613 (2014). 10.4103/1319-2442.128613 24626022

[25] Anees, M. *et al.* Demographic factors affecting quality of life hemodialysis patients. *Pak. J. Med. Sci.* **30**, 1123–1127. 10.12669/pjms.305.5239 (2014). 25225539 10.12669/pjms.305.5239 PMC4163245

[26] Manju, L., Joseph, J. & Beevi, N. Validation of kidney disease quality of life short form 36 (KDQOL-SF™) in malayalam among patients undergoing haemodialysis in South Kerala. *Indian J. Nephrol.* **30**(5), 316–320 (2020). 33707818 10.4103/ijn.IJN_139_19 PMC7869649

[27] Goh, K. K. K., Lai, P. S. M. & Lim, S. K. Cross cultural adaptation and validation of the Malay Kidney Disease Quality of Life (KDQOL-36™). *BMC Nephrol.* **20**(1), 226 (2019). 31221116 10.1186/s12882-019-1397-8 PMC6585031

[28] Bai, Y., Lai, L., Lee, B., Chang, Y. & Chiou, C. The impact of depression on fatigue in patients with hemodialysis: A correlational study. *J. Clin. Nurs.* **24**, 2014–2022. 10.1111/jocn.12804 (2015). 25827047 10.1111/jocn.12804

[29] Aydıner Boylu, A. & Paçacıoğlu, B. Yaşam kalitesi ve göstergeleri. *Akad. Araştırmalar Çalışmalar Dergisi* **8**, 137–150 (2016).

[30] Kring, D. L. & Crane, P. B. Factors affecting quality of life in persons on hemodialysis. *Nephrol. Nurs. J.* **36**, 15–24 (2009). 19271620

[31] Sağlık Teknolojisi Değerlendirme Daire Başkanlığı (STDD). *Böbrek Yetmezliği Tedavisinde Kullanılan Periton Diyalizi ve Hemodiyaliz Yönteminin Dolaylı Maliyetlerinin Analizi*. (Sağlık Araştırmaları Genel Müdürlüğü, 2017).

[32] Gökalp, K. & Arpacı, R. Diyaliz hastalarının psikolojik durumlarının değerlendirilmesi. *Turk. J. Sci. Health* **2**, 22–30 (2021).

[33] Fassbinder, T., Winkelmann, E., Schneider, J., Wendland, J. & Oliveria, O. Functional capacity and quality of life in patients with chronic kidney disease in pre-dialytic treatment and on hemodialysis—A cross-sectional study. *J. Bras. Nephrol.* **37**, 47–54. 10.5935/0101-2800.20150008 (2015). 10.5935/0101-2800.20150008 25923750

[34] Özşaker, E. Organ Nakli ve Yaşam Kalitesi. *Balikesir Saglik Bilimleri Dergisi* **3**, 166–173 (2014).

[35] Tung, H. H., Chen, H. L., Wei, J. & Tsay, S. L. Predictors of quality of life in heart-transplant recipients in Taiwan. *Heart Lung* **40**, 320–330. 10.1016/j.hrtlng.2009.11.003 (2011). 20561854 10.1016/j.hrtlng.2009.11.003

[36] Kaya, Ş *et al.* Böbrek Nakli Yapılan Hastalarda İdrar Yolu Enfeksiyonları: Sıklığı, Etkenler ve Risk Faktörleri. *Fırat Tıp Dergisi* **20**, 161–164 (2015).

