# Author Correction: Examinations of effects of socio-demographic features and disease-related data of patients with hemodialysis on the quality of life

**DOI:** 10.1038/s41598-025-87765-3

**Published:** 2025-01-30

**Authors:** Elif Togay, Hatice Öntürk Akyüz

**Affiliations:** 1 Department of Dialysis, Bitlis Tatvan State Hospital, Bitlis, Turkey; 2 Nursing Department, Faculty of Health Sciences, Bitlis Eren University, Rahva Campus, Beş Minare District, Ahmet Eren Boulevard, 13100 Center/Bitlis, Turkey

Correction to: *Scientific Reports* 10.1038/s41598-023-43473-4, published online 02 October 2023

The original version of this Article contained an error in the Abstract.

“The data collection process was performed face-to-face between 1 May and 30 June 2020.”

now reads:

“The data collection process was performed face-to-face between 15 March and 15 April 2021.”

The original Article has been corrected.

